# Causes and consequences of infections in patients after liver transplantation: 2-year study in the only ICU that hospitalizes these cases in Greece

**DOI:** 10.1186/cc13392

**Published:** 2014-03-17

**Authors:** A Karapanagiotou, C Kydona, S Papadopoulos, T Theodoridou, E Mouloudi, C Dimitriadis, G Imbrios, N Gritsi-Gerogianni

**Affiliations:** 1 Hippokrateion Hospital of Thessaloniki, Greece

## Introduction

Patients with liver transplantation suffer a major risk of infections immediately after the surgery due to heavy immunosuppression, severe stress and underlying pathology. The aim of this study is to find their causes andconsequences in our ICU.

## Methods

We studied 72 cirrhotic patients who were transplanted in our hospital during the years 2011 and 2012. The cases that developed intrahospital infection, its source and microbiology, the surgical complications, the function of the transplant, the duration of mechanical ventilation and their outcome were fully examined.

## Results

Twenty patients (27.1%) developed 33 episodes of infection. Among them 35% suffered from liver dysfunction, 40% took large doses of immunosuppression and 50% were re-operated. Five out of them showed signs of infection promptly after transplantation. Medium duration of mechanical ventilation was 16.5 ± 12.75 days (vs. 3.24 ± 5.5 of those without an infection) and medium length of stay was 22.14 ± 18.35 days (vs. 6.79 ± 8.86). The medium APACHE II score was 17.8 ± 5.5 (vs. 16.4 ± 4.37, *P *= 0.256) and medium SOFA was 11.32 ± 2.62 (vs. 9.9 ± 2.62, *P *= 0.054). The majority of patients was transfused with many blood units, FFP and cryoprecipitates and was hemodynamically unstable (56.3%). MELD score and MELD-Na were higher (22.57 and 27.5 relatively vs. 19.02 and 22.5). Eight cases had VAP from Gram- negative bacteria, 15 had bacteraemia (mainly Gram-negative, but also fungaemia), 10 had intraabdominal infection (most of them with two or three re-operations) and urine infection. The majority of pathogens were multidrug resistant. Regarding *Klebsiella pneumoniae *Carbapenemase, 60% was sensitive to colimycine, 20% to tobramycin, 70% to gentamycin and 40% to tygecycline. In four cases the donors had an infection that was transmitted to the recipient as much as 75%. Fourteen patients died (10 in ICU and four in the transplantation clinic) soon after with multiorgan failure.

## Conclusion

Patients after liver transplantation with organ dysfunction and multiple transfusions, due to surgical complications, have a major risk of developing an infection inside the ICU that affects their survival.

